# Closed non-suction drain placement as haematoma and seroma formation preventive measure post-nylon darn surgery for inguinoscrotal hernias in adults

**DOI:** 10.1007/s10029-021-02430-8

**Published:** 2021-06-11

**Authors:** Israel Hagbevor, Mahamudu Ayamba Ali, George Asare Awuku

**Affiliations:** 1 Surgical Unit, Margret Marquart Catholic Hospital, Kpando, Ghana; 2 Department of Surgery, School of Medicine, University of Health and Allied Sciences, Ho, Ghana; 3 Department of Medical Laboratory Sciences, School of Biomedical and Allied Health Sciences, University of Ghana, Korle, Bu, Accra, Ghana

**Keywords:** Non-suction, Drain, Nylon darn, Inguinoscrotal hernia

## Abstract

**Purpose:**

Inguinal hernia is a common male surgical disease. Intervention carries a wide range of complications such as scrotal haematoma and seroma which may require surgical re-intervention or predispose patients to developing infections, pains or feeling of mass. This could lead to long hospital stay. Scrotal tamponade by bandaging or wearing of tight pants and elevation are practiced to reduce bleeding and haematoma formation. These methods require prolong use. Closed suction drains are scarcely used in resource-deprived communities due to high cost and non-availability.

**Aim:**

This study was to determine the effectiveness of a closed non-suction drain in preventing scrotal collection requiring further surgical intervention and the predisposition to developing surgical site infection following nylon darn repair of inguinoscrotal hernia.

**Methods:**

Forty (40) participants were recruited for a preliminary study and assigned into control and interventional groups (CG, IG) for purposes of inserting flexible feeding tube (FFT) wound drain after nylon darn (ND) repair. Daily measurement of drained scrotal collection was carried out in the IG till the day drainage was zero. Residual volumes in IG and wound collection in the CG who were not candidates for re-intervention were determined at 14th and 28th post-operative days using ultrasound scan. Data were analyzed using SPSS version 25.

**Results:**

Three (3) patients (15.8%) in the CG required re-intervention. Surgical site infection rates for the CG and IG were, respectively, 2/19 versus 0/21 (*ρ* = 0.134).There was a numerical significant difference in the mean volumes of scrotal collections between the control (0.95 ± 0.42 ml) and the intervention group (0.44 ± 0.33 ml) [*p* value of 0.041] but with no clinical impact.

**Conclusion:**

Simple inexpensive flexible feeding tube placement significantly reduced scrotal collection which forms a base for larger sample size in subsequent studies. This could reduce the feared risk of re-intervention, wound infection and long hospital stay post-operative.

## Introduction

Inguinal hernia (IH) is a common surgical condition which affects males predominantly [1] with a prevalence ranging from 3.15 to 9.4% in Africa [2, 3]. In Ghana, about 12% of men with IH die without early surgical repair [4]. For the period 2015–2019, 60 (9.3%) out of the 643 IH cases managed at Margret Marquart Catholic Hospital (MMCH) presented with intestinal complications. The procedures for hernia represented about 59.1% of general surgical operations of MMCH, a percentage we believe is similar to most district hospitals in the country. Most patients with IH present with longstanding huge scrotal swellings which predispose them to post-op complications including scrotal collection [5, 6].

Nylon darn is an example of a tissue-based repair technique for IH. It is a tension-free approximation of the inguinal ligament to the conjoint tendon with nylon suture from the pubic tubercle to the internal ring. This weave in the posterior wall of the inguinal canal was first described by Moloney. This method was chosen based on competence, cost effectiveness and its relative good potential to withstand surgical site infection [7].

Scrotal haematoma and seroma are common post-operative complications thought to result from the extensive sac separation in huge and long standing indirect inguinal hernias with reported incidence of 8–22% [8, 9].

Warfarin usage, valvular heart disease, atrial fibrillation, hypertension, recurrent hernia and coronary artery disease are also significant pre-operative risk factors for hematoma formation post IH repair, even though warfarin usage and recurrent hernia are independent risk factors [10]. Scrotal haematoma and seroma predispose patients to developing surgical site infection (SSI) as the collection can form a good culture medium for inoculated bacteria growth. SSI may require additional surgical intervention when antibiotic treatment fails. Other consequences of SSI include prolonged hospital admission, weak and ugly scar formation and hernia recurrence.

**Fig. 1:**
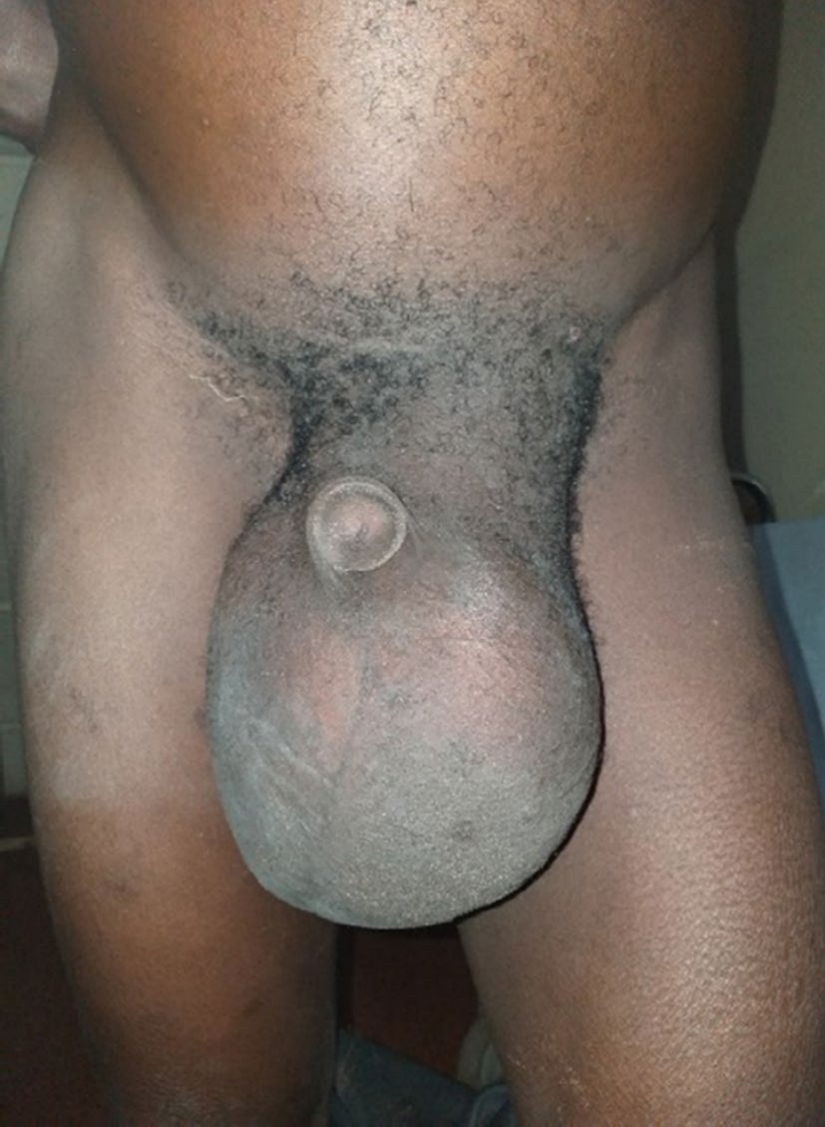
A huge reducible left complete inguinoscrotal hernia

**Fig. 2:**
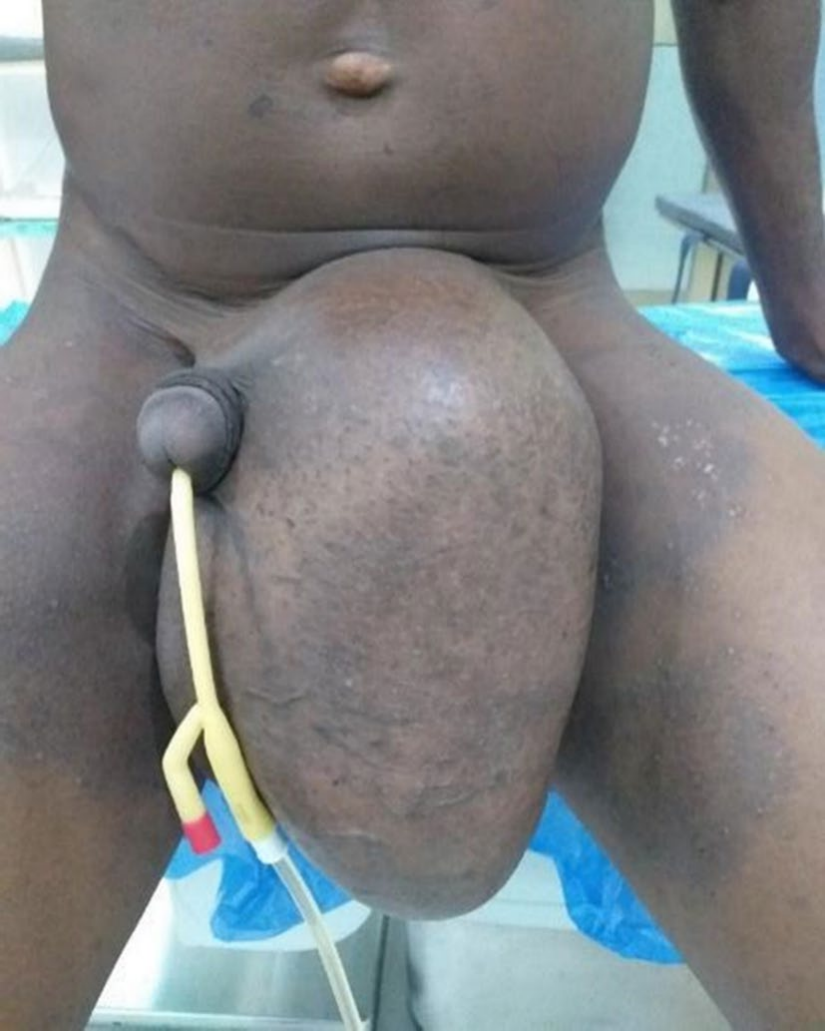
An obstructed left complete inguinoscrotal hernia

Figures 1 and 2 are examples of hernia types commonly managed in MMCH.

Post-surgical scrotal bandaging and or elevation, wearing of tight pants and closed-suction drains have been in practice to minimize bleeding and or accumulation of blood at surgical sites which are either expensive or ineffective [11].

Treatment of large inguinoscrotal hernias has specific aspects that are challenging in an environment where guidelines cannot always be followed and financial barriers for standard care are high. It is relevant to investigate the best and safest manner to treat inguinoscrotal hernias. Very little is written about non-suction drains which may effectively decrease post-op seroma/haematoma formation.

The aim of the study was to determine the usefulness of inserting a flexible feeding tube as a non-suction wound drain in preventing any blood and seroma accumulation at surgical site and scrotum therefore reducing the incidence of re-intervention, patient anxiety, wound infection and further antibiotic treatment.

### Outcome measures

The main outcome was a need for surgical re-intervention for scrotal collection as stated in Table 1.

**Table 1:** Outcome measures of the study

Type of outcome	Outcome measures	Duration
Primary outcome	1. The need for re-intervention	Assessed for 1 month post-op
Secondary outcome	1. Surgical site infection 2. Post-operative pain from drain site	Was assessed first 5 days post-op Was assessed 24–48 h post-op

Evaluation for surgical site infection was done using clinical signs: erythema, wound discharge, wound gape. Suspected sites of infection were swabbed for culture and sensitivity testing.

Post-operative pain was assessed using Numeric Rating Scale. All participants were requested to give an answer at post-operative days 1 and 2.

### Terminologies

For the purposes of this study, scrotal seroma is defined as any volume of fluid accumulation in the scrotum post-operation readily visible on physical examination and of concern to the patient.

Scrotal haematoma is also defined as the accumulation of blood of any volume in the scrotum post-operation which is readily visible on physical examination and of concern to the patient.

Scrotal collection refers to haematoma and or seroma.

## Materials and methods

This was a preliminary study involving forty (40) adult males with complete inguinoscrotal hernia (Miserez type C hernias or H3–H4 hernias) who sort nylon darn repair at MMCH, in the Volta region of Ghana. The diagnosis of complete inguinoscrotal hernia was made preoperatively through clinical assessment and confirmed intra-operatively by an existing sac which was completely separated from the spermatic cord structures as described by BAJA [12].

The forty (40) participants were randomly assigned to intervention and control groups (21:19), respectively. All team members were pre-trained on the protocols of the study. None bias was maintained by a pre-determined order of assigning patients to the groups which was handled by a ward nurse who was not part of the operating team. The other team members comprising anaesthetists and the theatre nurses were also blinded until the need for insertion of the FFT was communicated to them in theatre.

### Inclusion criteria

Complete inguinoscrotal hernia patients aged 18 years and above at the surgical outpatient department and emergency units who were diagnosed and clinically evaluated through history to identify comorbidities or potential cause of bleeding disorder were included in the study.

### Exclusion criteria

Patients who did not complete the follow up instructions were excluded from the study. Furthermore, those in the intervention group whose flexible feeding tube fell off before time for drain removal were excluded in addition to participants who opted out of the study.

### Study design

Physical examination was carried out to identify any ecchymosis, petechiae, haemolysis with jaundice and general fitness of the patient for surgery. Laboratory investigations included Full blood count indexes to ensure that the pre-operative haemoglobin level, leucocytes and differential counts and platelet levels were within normal range. Bedside clotting time and other necessary investigations such as chest X-rays, electrocardiogram (ECG), urinalysis were also done to ensure patients’ optimization. The standard protocol for nylon darn repair was explained to all patients as well as available options for scrotal support post-operative and their expected outcomes. Those who were willing to participate in the study were taken through the protocol to their understanding. The information included an insertion of a secured flexible feeding tube connected to a collecting bag at the wound site as a drain which was to be maintained till the principal investigator instructed its removal. Any collection in the drain bag was emptied and measured by the investigator. All clinical changes such as bleeding, fever, pains, discharge from wound, etc. observed must be reported for immediate evaluation apart from the routine scheduled ward rounds during the period of the study. They were also made aware of the requirement to return after discharge from hospital for scrotal ultrasound scan on post-operative day 14 and 28. An informed consent was then obtained from the willing participants (WP) and the ward nurse assigned a consecutive count each time a participant was recruited starting from 1. All odd numbered WP were pre-determined to belong to the control group (CG), while even numbered WP were assigned interventional group (IG). This order was maintained throughout the study independent of any influence.

A structured questionnaire, partly open-ended was administered for demographic data of WP prior to surgery. Participants then started our routine pre-operative counselling and anaesthesia review.

The pre-trained nurses at the surgical ward verified anytime a recruited participant was admitted to the ward after going through the study protocol, understood the aim, expectation of the study and if patient had any concerns regarding his participation. The theatre assistant verified the assigned number and made the flexible feeding tube and collecting bag ready for any IG participant who was brought to theatre for the procedure. They were also always reminded by the theatre assistant the right to withdraw anytime the tube was shown to them.

### Surgical procedure

Standard prophylactic antibiotic was given to all patients per the hospital protocol and continued in selected patients when it was necessary per the intra-operative findings. Participants had either local, spinal or general anaesthesia based on the independent judgment of the anaesthesia team.

**Fig. 3:**
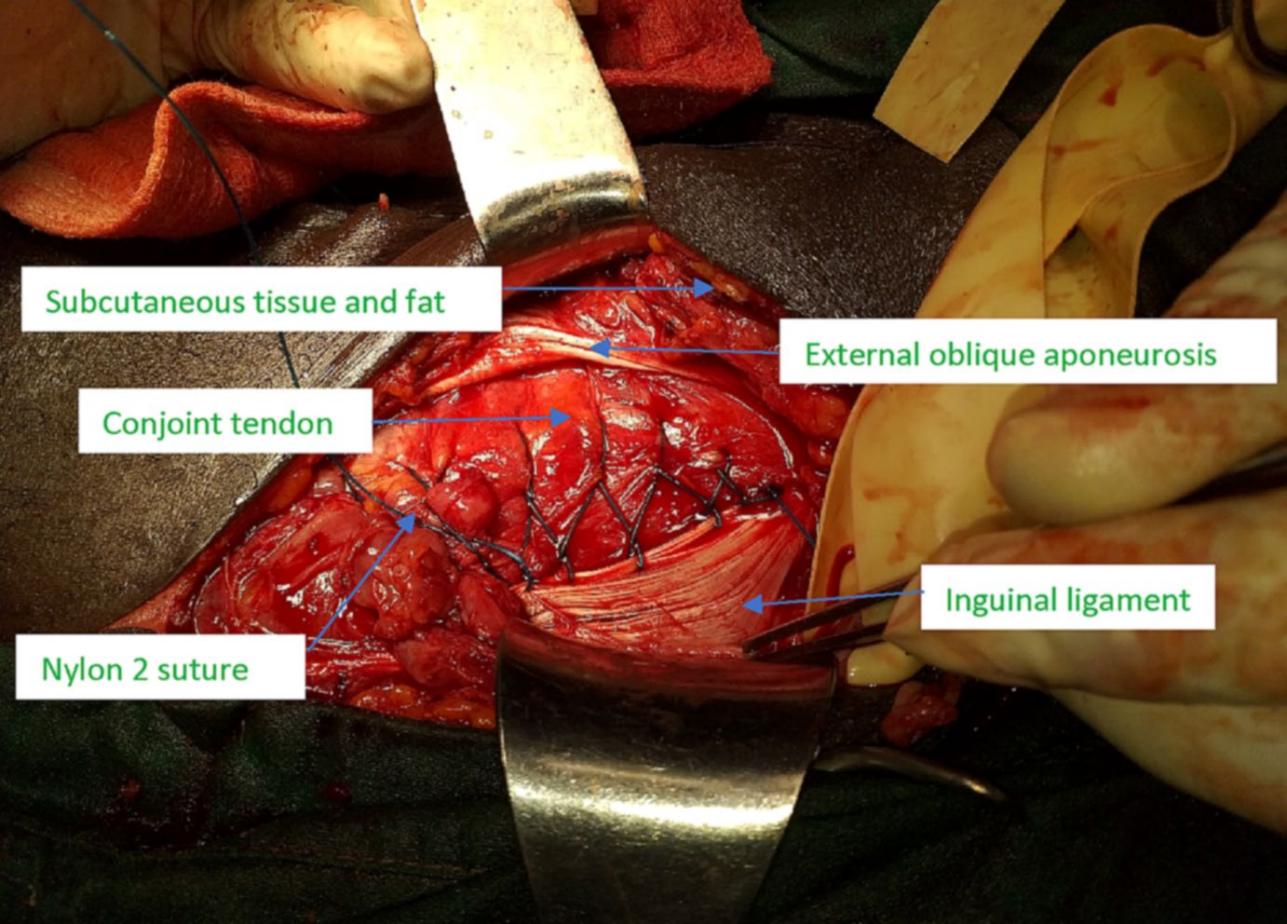
Nylon darn repair of a posterior wall

The hernia sac separation and posterior wall re-enforcement was done using nylon darn technique described by Moloney (Fig. 3).

### Intervention

**Fig. 4:**
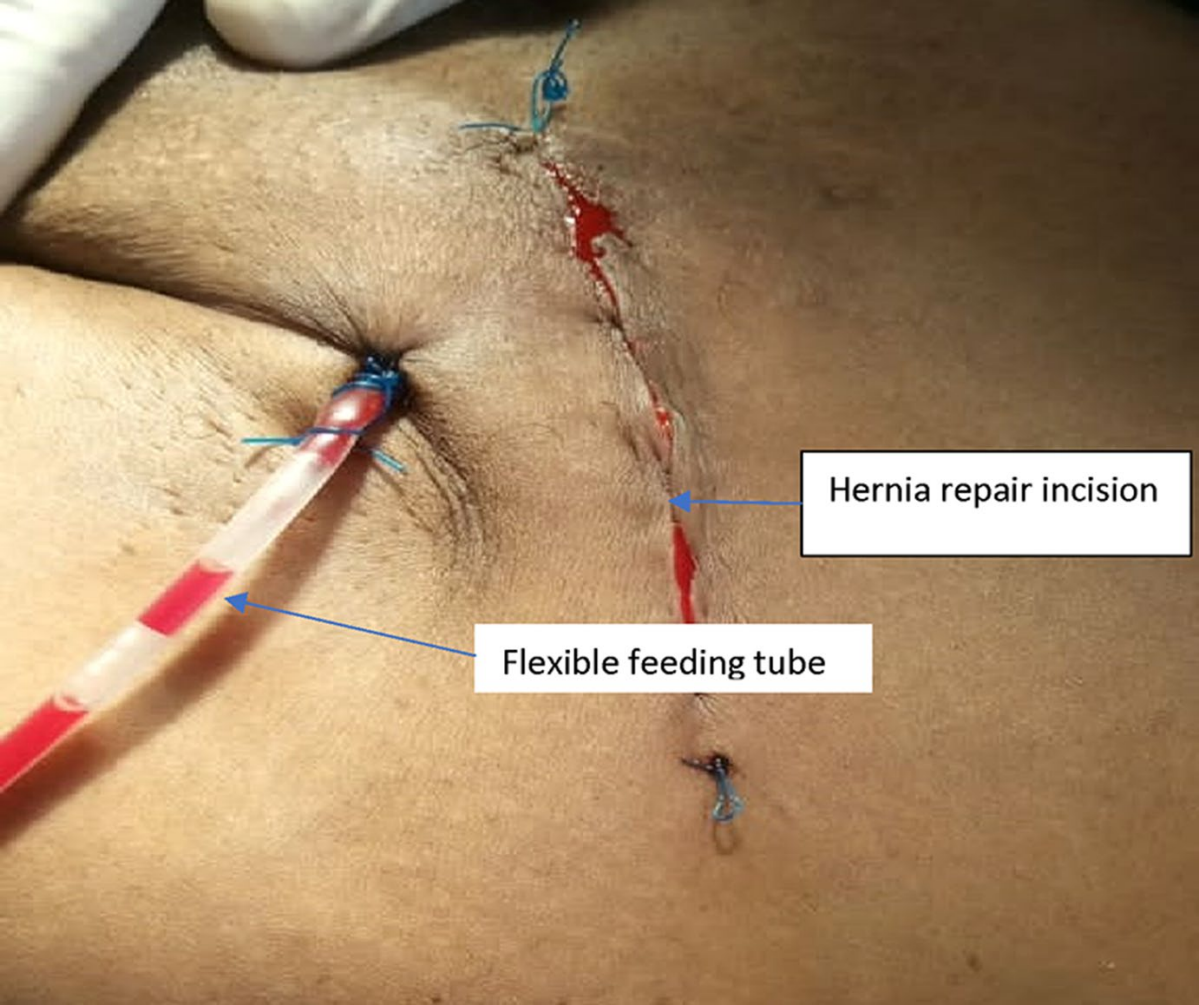
The right groin showing inserted flexible feeding tube

Following closure of the external oblique aponeurosis, the FFT connected to a collecting bag at an opposite end was advanced to the scrotum as described by Narayanswamy et al. [13] and secured to the skin with nylon 2/0. Volumes of fluid drained by intact tubes and bags in the IG were determined post-operatively after carefully emptying it into graduated measuring jar daily (Fig. 4).

The drain was removed when it remained empty for 24 h. Scrotal ultrasound scan was carried out in both groups on post-operative days 14 and 28 to measure volumes of scrotal collections.

After each procedure, the pain management was the same for all participants as recommended by the hospital’s protocol.

The mean volumes of scrotal collections in IG and CG measured by ultrasound scan were compared using independent sample *t* test. Other demographic characteristics were analyzed with descriptive statistics. Daily clinical review for scrotal swelling and the need for surgical evacuation were done. Inspection for surgical site infection and the need to swab wound for culture and sensitivity test and institution of antibiotic treatment were also done.

## Results

The forty (40) participants aged 18–82 (46.3 ± 17.1) years. The mean ages of the CG and IG were 48.9 ± 17.4 years and 44.0 ± 16.9 years, respectively. The highest incidence of hernias occurred between 48 and 57 years with no significant difference between the two groups.

One (4.8%) participant in the IG had diabetes mellitus. Six (31.6%) in the CG and 3 (14.3%) participants of the IG were hypertensive.

Majority (52.5%) of participants had hernia for 1–5 years and 4 (10%) had the disease for over 10 years, while 3 (7.5%) of the participants had congenital hernia. The average duration of participants’ hernia was 3.2 ± 1.0 years with the IG and CG averages 3.0 ± 0.9 and 3.4 ± 1.1 years, respectively (ρ > 0.05) (Table 2).

**Table 2:** Patient characteristics

Variable	Control group (*n* = 19) *n* (%)	Intervention group (*n* = 21) *n* (%)	*p* value
Average age (years)	48.9 ± 17.4	44.0 ± 16.9	0.203
Medical condition			
Hypertension	6 (31.6)	3 (14.3)	> 0.05
Diabetes mellitus	0 (0.0)	1 (4.8)	> 0.05
Location of hernia			> 0.05
Left	4 (21.1)	5 (23.8)	> 0.05
Right	15 (78.9)	16 (76.2)	
Duration of hernia			
< 1 year	2 (10.5)	2 (9.5)	
1–5 years	8 (42.1)	13 (61.9)	
6–10 years	5 (26.3)	3 (14.3)	
> 10 years	3 (15.8)	1 (4.8)	
Congenital	1 (5.3)	2 (9.5)	
Nature of hernia at presentation			0.036
Reducible	13 (68.4)	14 (66.7)	
Obstructed	6 (31.6)	7 (33.3)	
Content of hernia sac			
Viable small bowel	4 (21.1)	7 (33.3)	
Viable caecum	0 (0.0)	4 (19.1)	
Gangrenous caecum	1 (5.3)	2 (9.5)	
Gangrenous small bowel	2 (10.5)	0 (0.0)	
Empty sac	12 (63.2)	8 (38.1)	

The commonest cause of delay in seeking surgical intervention was financial constraint (75.0%) followed by preference for herbal medicine (45.0%) and fear of surgery (35.0%).

Thirteen (32.5%) presented with intestinal complications with three having resection for gangrenous caecum and 2 gangrenous small bowel. The other eight had intestinal obstruction with viable bowel in the sac, four each for small and large bowel.

Most participants 27 (67.5%) had a reducible hernia. Majority (50.0%) of the participants presenting for elective hernia repair had empty hernia sac, while 17.5% had viable small bowel as the hernia sac content. Spinal anaesthesia was used in 31(77.5%) and local infiltration for 6 (15.0%) of the participants. Three (7.5%) underwent general anaesthesia.

The mean length of drains inserted was 15.2 ± 5.8 cm. Majority of the participants (76.2%) in the IG had their wound drain in-situ for 2 days and 19.0% had it in-situ for 3 days. The duration of wound drain ranges between 1 and 3 days with a mean of 2.1 ± 0.5 days. The measured volumes of all collections drained in the intervention group range between 3.5 and 190 ml with a mean of 33.4 ± 26.5 ml.

Regarding the level of restriction of movement with the inserted drain, only one patient (4.8%) reported moderate restriction of his movement due to the presence of the inserted drain. Six (28.5%) experienced mild restriction, while majority (66.7%) did not report any restriction at all.

The incidence of scrotal haematoma/seroma between the CG and IG was (4/19) 21.1% and 0.0%, respectively (ρ = 0.027). Three (15.8%) of them required re-intervention (evacuation) with drain insertion and wound closure. The volume of evacuated scrotal haematoma ranges between 350 and 400 ml with a mean of 384.3 ± 17.9 ml (Table 3).

**Table 3:** Post-operative complications

Complication	Control group (*n* = 19) *n* (%)	Intervention group (n = 21) *n* (%)	*p* value
Scrotal seroma/haematoma	4 (21.1)	0 (0.0)	0.027
Scrotal oedema	3 (15.8)	2 (9.5)	0.561
Surgical site infection	2 (10.5)	0 (0.0)	0.134

Surgical site infection occurred in 2 participants of the CG who had small bowel resection and anastomosis and none in IG (ρ = 0.134). Blood culture in both participants isolated *Citrobacter koseri* species which was sensitive to Amikacin and Ciprofloxacin.

Scrotal oedema occurred in 3 (15.8%) CG and 2 (9.5%) IG (*p* = 0.561). No mortality was recorded in this study.

Participants of the IG stayed between 2 and 6 days with a mean duration of 2.5 ± 1.0 day, while those of the CG stayed between 1 and 34 days with a mean of 8.2 ± 6.3 days. The three participants who underwent haematoma evacuation stayed for 10, 11 and 14 days each. Those who had surgical site infection also stayed for 19 and 34 days before discharged from hospital (Table 4).

**Table 4:** Ultrasound measured scrotal collections among the participants

Study groups	Drain inserted *n* = 21		No drain inserted *n* = 16		Total *N* = 37		
	Mean	SD	Mean	SD	*t*-value	df	*p* value
Scrotal collections	0.44	0.33	0.95	0.42	0.837	7	0.041

The volumes of ultrasound measured scrotal collections in the CG (excluding three participants that underwent haematoma evacuation) ranges between 0.8 and 28.9 ml with a mean volume of 0.95 ± 0.42 ml and that of the IG was between 0.0 and 5.8 ml with a mean of 0.44 ± 0.33 ml which was statistically significant (*p* 0.041).

## Discussion

Hernia repair as with other surgical procedures is not without complications. One of the commonest complications following inguinal hernia repair is scrotal seroma and haematoma formation [9, 10, 14]. These scrotal collections do not only affect the patients psychologically but also could act as suitable medium for inoculated bacterial growth with resulting surgical site infection and its attendant complications. Continuous research that seeks the use of cost effective and readily available biomaterial to prevent collections is useful especially among trainee surgeons.

Several factors such as warfarin use, hypertension, atrial fibrillation, valvular heart disease, recurrent hernia and coronary artery disease have been identified as significant pre-operative risk factors [10]. Most of these likely confounders of post-operative haemorrhage such as bleeding disorders and use of blood thinners such as warfarin, aspirin and clopidogrel have been excluded among the participants. The other modifiable risk factors such as hypertension and diabetes mellitus have been appropriately managed to eliminate their influence on the outcome of this study.

Hypertension, an important risk factor for post-operative scrotal haematoma formation was a comorbidity in 9 willing participants. However, these patients had achieved good blood pressure (BP) control (BP < 140/90 mmHg) on oral antihypertensives prior to and after the surgery. Its influence to formation of haematoma or seroma was not statistically significant between the two groups (*ρ* > 0.05).

Intestinal complication of hernia posed a significant risk to scrotal haematoma, seroma formation and surgical site infection [15]. Elective hernia repair was carried out in majority (67.5%) of the participants which is consistent with general trend in most studies [16]. Six (31.6%) control and 7 (33.3%) intervention group participants underwent an emergency hernia repair with varied hernia sac contents. The contents of the hernia sac invariably affected the risk of infection as well as scrotal collection formation.

Surgical site infection occurred in 2 out of the 19 (10.5%) participants in the control group, while none was observed in the intervention group despite the resection and anastomosis of gangrenous bowel segments in both groups. Indeed, the less than 1 ml ultrasound measured scrotal collection difference between the two groups may appear irrelevant. However, regarding risk of surgical site infection, the least volume of scrotal collection that may occur in the presence of bowel content contamination at time of surgery may significantly increase the risk of surgical site infection and its attendant complications. This therefore buttresses the point that near zero scrotal collection post-operative should always be aimed at. SSI may require additional surgical intervention when antibiotic treatment fails.

In addition, while no participant in the intervention group developed scrotal haematoma/seroma, four (4) (21.1%) of the participants in the control group developed scrotal haematoma post-operative (*p* < 0.05). The scrotal collections were significant among three (15.8%) of the participants necessitating a re-intervention, an outcome which the use of the drain is intended to avoid. It was observed that all three participants who underwent re-intervention had an emergency hernia repair with one requiring bowel resection and anastomosis for a gangrenous caecum.

Length of hospital stay was reduced significantly in the intervention group (2–6 days with a mean of 2.5 ± 1.0 days) compared to the control group (1–34 days with a mean of 8.2 ± 6.3 days) (*p* = 0.012). This intervention had demonstrated effectiveness in significantly reducing the risk of re-intervention, length of hospital stay, and surgical site infection especially during emergency repair of huge inguinoscrotal hernias.

## Conclusion

The function of flexible feeding tube as a closed non-suction drain in this preliminary study provided a promising alternative scrotal collection preventive measure after inguinoscrotal hernia repair with nylon darn without side effects. Furthermore, this will be readily available, accessible to low income and developing regions.

## Data Availability

The data that support the findings of this study are available from the corresponding author, [I.H], upon reasonable request.

## Appendix 1 Materials used for the surgery

**Table:**

Material	Description
Sutures	Round bodied ½ C 40 mm Nylon 2, Reverse cutting ½ C 31 mm Nylon 2/0 and Round bodied ½ C 40 mm Polyglycolic acid Violet 2. All manufactured by Shangai Channelmed Import & Export Co, Ltd, China
Drain	Sterile non-toxic stomach tube, 16 FR, 49-inch-long. Manufactured in China and supplied by SHANGHAI HESN IMP & EXP.CO LTD

## Code availability

The custom code of this research is available from the corresponding author, [I.H] upon reasonable request.
